# Positive Association of Urinary Dimethylarsinic Acid (DMA^V^) with Serum 25(OH)D in Adults Living in an Area of Water-Borne Arsenicosis in Shanxi, China

**DOI:** 10.3390/toxics12010083

**Published:** 2024-01-18

**Authors:** Kunyu Zhang, Yunyi Yin, Man Lv, Xin Zhang, Meichen Zhang, Jia Cui, Ziqiao Guan, Xiaona Liu, Yang Liu, Yanhui Gao, Yanmei Yang

**Affiliations:** 1 Center for Endemic Disease Control, Chinese Center for Disease Control and Prevention, Harbin Medical University, Harbin 150081, Chinayinyunyi@hrbmu.edu.cn (Y.Y.);; 2 Key Laboratory of Etiology and Epidemiology, Education Bureau of Heilongjiang Province & Ministry of Health of P. R. China, Harbin Medical University, Harbin 150081, China

**Keywords:** arsenic, serum 25(OH)D, methylation capacity, skin hyperkeratosis

## Abstract

Limited studies have demonstrated that inorganic arsenic exposure is positively associated with serum vitamin D levels, although the correlation between urinary arsenic species and serum vitamin D has not been investigated in areas of water-borne arsenicosis. A cross-sectional study of 762 participants was conducted in Wenshui Country, Shanxi Province, a water-borne arsenicosis area. The results showed a positive relationship between urinary arsenic species (inorganic arsenic (iAs), methylarsonic acid (MMA^V^), dimethylarsinic acid (DMA^V^) and serum 25(OH)D. Log-binomial regression analysis indicated a 0.4% increase in the risk of vitamin D excess for every 1-unit increment in the Box–Cox transformed urinary DMA^V^ after adjustment for covariates. After stratifying populations by inorganic arsenic methylation metabolic capacity, serum 25(OH)D levels in the populations with iAs% above the median and primary methylation index (PMI) below the median increased by 0.064 ng/mL (95% CI: 0.032 to 0.096) for every one-unit increase in the Box–Cox transformed total arsenic (tAs) levels. Serum 25(OH)D levels increased by 0.592 ng/mL (95% CI: 0.041 to 1.143) for every one-unit rise in the Box–Cox transformed iAs levels in people with skin hyperkeratosis. Overall, our findings support a positive relationship between urinary arsenic species and serum 25(OH)D. It was recommended that those residing in regions with water-borne arsenicosis should take moderate vitamin D supplements to avoid vitamin D poisoning.

## 1. Introduction

Vitamin D is known for its role in supporting bone growth [1]. Some studies have also shown beneficial links between vitamin D and other non-skeletal health outcomes, including immune function, cardiovascular health, and cancer [2,3]. Vitamin D acts in the body through both an endocrine mechanism (regulating calcium absorption in the gastrointestinal tract) and an autocrine mechanism (facilitating the expression of CYP27B1, CYP24A1, and vitamin D receptor in bone) [4,5]. However, both vitamin D deficiency and excess can lead to serious health problems. Vitamin D deficiency is the most common nutrient deficiency worldwide [6]. It is associated with a number of disorders such as metabolic, autoimmune, psychiatric, cardiovascular diseases, cancer (particularly colon, prostate and breast cancer), and chronic pain disorders [7,8,9,10]. Vitamin D, a fat-soluble steroid hormone, can also cause serious health problems if taken in excess. Early symptoms of vitamin D toxicity include drowsiness, persistent headache, irregular heartbeat, and loss of appetite [4,11].

The synthesis, absorption and metabolic processes of vitamin D can be influenced by many factors, including environmental factors such as sun exposure, latitude and season, as well as individual factors such as obesity, genetics, skin complexion and age [12,13]. It is also suggested that environmental pollutants can affect the biosynthesis of vitamin D precursors on the metabolism of vitamin D [14]. Epidemiological studies suggest an association between air pollution and vitamin D deficiency [15]. In cities with high levels of air pollutants (ozone, particulate matter and sulfur dioxide), ultraviolet light is efficiently absorbed by these pollutants, reducing skin synthesis of pro-vitamin D3 [16]. Since toxic metals such as cadmium (Cd) and lead (Pb) are widespread in the environment and may play a role in endocrine disorders, it is necessary to investigate the effects of exposure to toxic metals on human vitamin D status [14,17]. Some population-based studies have shown that Cd and Pb can affect vitamin D status in humans [18,19,20]. Several studies conducted in Cd-contaminated areas of Japan found that serum vitamin D levels were lower in Cd-exposed elderly subjects (Cd-exposed man: mean urine Cd = 12.1 μg/g creatinine; Cd-exposed woman: mean urine Cd = 16 μg/g creatinine) than in unexposed older subjects [18,19]. As an endocrine modulator in the human population, Pb is also considered one of the risk factors for vitamin D deficiency. A cohort study of pregnant women showed that vitamin D intake higher than 5.4 μg per day was negatively correlated with Pb in maternal blood and Pb in cord blood [20]. Another cross-sectional study showed a significant negative correlation between blood Pb levels and serum 1,25(OH)_2_D in children exposed to Pb [21].

Inorganic arsenic, a known environmental pollutant, is listed first on the Hazardous Substances List by the Agency for Toxic Substances and Disease Registry (ATSDR) [22]. Arsenic is widely distributed in environmental media such as air, water, and soil [23]. It is worth noting that water-borne inorganic arsenic contamination has become a major human health problem worldwide [24]. Long-term drinking of water containing excessive inorganic arsenic can lead to liver and kidney damage, cardiovascular disease, diabetes and cancer [23]. Similar to heavy metals such as Cd and Pb, inorganic arsenic can affect vitamin D status [25,26,27]. It was found that As^III^ increased the transcriptional activity of paricalcitol, a non-calcemic vitamin D analogue in humans, probably by reducing the function of the mitochondrial enzyme 24-hydroxylase, which is responsible for the metabolism of active vitamin D in acute myeloid leukemia cells (AML) [25]. A cross-sectional study conducted near a Pb smelter in the city of Torreón, Mexico, found positive associations between urinary total arsenic and serum 1,25(OH)_2_D, but no association between urinary total arsenic and serum 25(OH)D [26]. Another prospective hospital-based cohort study (598 study subjects) focused on the associations of 20 metals (Pb, Cd, aluminum, vanadium, chromium, manganese, cobalt, nickel, copper, zinc, arsenic, selenium, rubidium, strontium, argentum, cesium, barium, thallium, thorium and uranium) during pregnancy with umbilical cord serum vitamin D levels and found that urinary total arsenic was positively associated with serum 25(OH)D [27]. However, no studies have been conducted on the association between urinary arsenic species and serum 25(OH)D in areas with water-borne arsenicosis.

The key factor in a person’s susceptibility to inorganic arsenic is the ability to metabolize and efficiently excrete inorganic arsenic [28]. After human absorption, As^III^ accepts a methyl group from S-adenosylmethionine and is methylated to methylarsonic acid (MMA^V^) or dimethylarsinic acid (DMA^V^) [24,29]. MMA^V^ is then reduced to methylarsonous acid (MMA^III^) and is oxidatively methylated to DMA^V^ [30]. Dimethylarsinous acid (DMA^III^) is the proposed essential intermediate in the methylation of DMA^V^ to trimethylarsenic oxide (TMA^V^O) [31]. In human urine samples, MMA^V^ and DMA^V^ are the main metabolites of inorganic arsenic exposure due to the low stability of MMA^III^ and DMA^III^ [30,32,33]. Efficient methylation of As^III^ to DMA^V^ is associated with lower health risks related to inorganic arsenic and increased excretion of inorganic arsenic compared to those with a lower methylation capacity [34]. The extent of As^III^ methylation varies within and between individuals and populations, suggesting that inorganic arsenic metabolism is influenced by genetic components [35,36]. However, it is still unknown whether inorganic arsenic metabolic capacity [urinary iAs%, urinary MMA^V^%, urinary DMA^V^%, primary methylation index (PMI), and secondary methylation index (SMI)] can influence the association between arsenic and 25(OH)D levels. PMI and SMI are important indicators for evaluating the level of arsenic methylation and both are calculated based on the arsenic species that are present in urine. Higher PMI and SMI suggest that arsenic methylation is more adequately metabolized and inorganic arsenic residues will be less [37].

As we know, skin is the only organ that can produce vitamin D [38]. Most vitamin D is obtained by irradiating the skin with ultraviolet B (UVB), which stimulates the conversion of 7-dehydrocholesterol to vitamin D_3_ (cholecalciferol) [39,40]. The skin is also a toxic target organ for inorganic arsenic. Hyperpigmentation and hyperkeratosis of the skin have long been recognized as typical signs of chronic exposure to inorganic arsenic [41]. Therefore, the association of inorganic arsenic with vitamin D in a population with skin lesions requires further investigation.

Serum 25(OH)D is considered a reliable marker of vitamin D deficiency because it has the highest abundance of all vitamin D metabolites in serum and its level remains stable for almost two weeks [6,42]. In the present study, we analyzed the relationship between urinary arsenic species and the ability of inorganic arsenic metabolism to serum 25(OH)D. We also examined whether skin keratinization induced by inorganic arsenic exposure affects the relationship between urinary arsenic species and serum 25(OH)D.

## 2. Materials and Methods

### 2.1. Study Site and Population

Data for this study were obtained from a cross-sectional study on the association between inorganic arsenic exposure and diabetes conducted in 2019 in Wenshui County, Shanxi Province, People’s Republic of China, which was identified as an area of water-borne inorganic arsenic exposure area according to long-term monitoring data provided by the Shanxi Institute of Endemic Disease Prevention and Control. All participants were given face-to-face questionnaires and a general physical examination by well-trained staff. General demographic data included general demographic characteristics (age, gender, height and weight), socioeconomic status (education and occupation), and milk consumption (≤1 glass/week and >1 glass/week), as milk is rich in vitamin D. We excluded 12 participants who had consumed seafood in the past 2–3 days to rule out the interference of arsenobetaine in urinary tAs levels, since arsenobetaine is a non-toxic arsenic species present in the urine of people who eat seafood. Participants’ body mass index (BMI) was also calculated based on height and weight. Skin hyperkeratosis (a hyperkeratotic thickening of the skin typically on the palms and soles) was ascertained by diagnosis of endemic arsenism (WS/T 211-2015) by study physicians. In total, 762 participants were included in the final analysis after excluding participants with incomplete baseline information and abnormal liver and kidney function. This study was approved by the Ethics Committee of Center for Endemic Disease Control, Chinese Center for Disease Control and Prevention, Harbin Medical University. The protocol code is HRBMUECDC20210410.

### 2.2. Sample Collection

Blood and urine samples from the participants were collected by professional nurses at the Affiliated Hospital of Shanxi Institute of Endemic Disease Prevention and Control. The blood used in the study was collected from cubital fossa veins using EDTA anticoagulated tubes (Beideng Medical Co., Harbin, China, Cat. No. V253489). After standing for 2 h, serum and blood cells were centrifuged in place (3000 rpm for 10 min). The instant urine was collected and divided into 1.5 mL Eppendorf (EP) tubes (Corning Ltd., New York, NY, USA, Cat. No. MCT-150-C) after centrifugation (3000 rpm for 10 min). Samples were frozen and stored in a refrigerator at −80 °C until analysis.

### 2.3. Determination of Urinary Arsenic Concentrations

The determination of arsenic species was performed by high-performance liquid chromatography–hydride generation–atomic fluorescence spectrometry (HPLC-HG-AFS) (LC-AFS 6500, Haiguang Instrument Co., Ltd., Beijing, China) after the collected urine samples were stored in the −80 °C refrigerator for approximately 4 months. In detail, arsenic species were separated by a Hamilton PRP-X100 (250 mm × 4.1 mm, 10 μm, Hamilton Laboratory Equipment Co., Ltd., Shanghai, China) column with a mobile phase consisting of 13 mmol/L CH_3_COONa (analytical grade, Sinopharm Chemical Reagent Co., Shanghai, China, Cat. No. C21266), 4 mmol/L KNO_3_ (analytical grade, Sinopharm Chemical Reagent Co., Shanghai, China, Cat. No. 10017218), 3 mmol/L NaH_2_PO_4_ (analytical grade, Tianjnin Guangfu Fine Chemical Research Institute, Tianjin, China, Cat. No. 13472-35-0), and 0.2 mmol/L EDTA-2Na (analytical grade, Aladdin Biochemical Technology Co., Shanghai, China, Cat. No. E116428) at a flow rate of 1.0 mL/min. The PH of the mobile phase was 6.0. The mobile phase was filtered through a 0.45 µm mixed cellulose ester (MCE) membrane filter (Biosharp Biotechnology Co., Hefei, China, Cat. No. BS-M-MCE13-45) and degassed by ultrasonication for 30 min before use. The injection volume was 100 µL and the argon pressure was 0.3 MPa. The liquid-phase high-pressure conditions were as follows: a column temperature of 30 °C; an upper pressure limit of 40 MPa; a lower pressure limit of 0 MPa. The atomic fluorescence conditions were as follows: a main current of 70 mA; auxiliary current of 35 mA; carrier gas flow of 300 mL/min; shielding gas flow of 900 mL/min; negative high pressure of 310 V; pump speed of 65 rpm. The wavelength of the sensitive line was 193.7 nm. The column effluent was delivered to a three-way cock and first mixed with 10% HCl (analytical grade, Huanghua City Century Kobo Science and Technology Development Co., Hebei, China) and then reacted with 3% KBH_4_ (analytical grade, Tianjin Komeo Chemical Reagent Co., Tianjin, China) in 0.5% KOH (analytical grade, Tianjnin Guangfu Fine Chemical Research Institute, Tianjin, China) in another three-way cock, where hydride generation took place. The concentrations of the arsenic compounds were expressed as concentrations of the arsenic element (As). The arsenic standards (National Institute of Metrology. China, Cat. As^III^: GBW08666, As^V^: GBW08667, MMA^V^: GBW08668, DMA^V^: GBW08669) were used to assess the accuracy during analysis. If the arsenic content in the urine form of the sample is lower than LOD, the value is LOD/$\sqrt{2}$. Atomic Fluorescence Speciation Analyzer Software (Version 2.0.01, Beijing Haiguang Instrument Co., Ltd., Beijing, China) was used to integrate the arsenic peaks. This software features the calculation function that can automatically search for arsenic peaks in each chromatogram and calculate peak areas. All arsenic peaks were identified using the retention time of reference standards of arsenicals. The calibration curves of arsenicals were fitted using peak areas and corresponding arsenical standard concentrations. The concentrations of each arsenic species were calculated by means of the peak areas and the corresponding calibration curves.

### 2.4. Determination of Serum 25(OH)D Concentrations

Serum 25(OH)D was detected using the biochemical analyzer 7180 (Hitachi High-Technologies Corporation, Tokyo, Japan). The vitamin D status of participants was categorized according to the definition of the 25 hydroxyvitamin D test kit (Meikang Biotechnology Co., Ningbo, China, Cat. No. 454), which was as follows: 25(OH)D < 20 ng/mL was defined as deficient, 21–29 ng/mL as inadequate, 30–100 ng/mL as adequate and >100 ng/mL as excessive.

### 2.5. Determination of Blood Glucose Concentrations

The participants were asked to fast overnight. Whole blood glucose was measured on the day of blood collection using a glucose tester (Acon Biotechnology Co., Hangzhou, China).

### 2.6. Quality Assurance and Quality Control

In order to train the investigators and standardize the survey methods according to the relevant content of the questionnaire, a training manual and a working manual were developed. Frozen human serum samples were equilibrated at room temperature for 30 min before use and then centrifuged at 12,000× *g* for 5 min to filter out precipitate. In addition, 1 mL of urine stored at −80 °C was thawed at room temperature and centrifuged at 13,000 rpm for 5 min. Urine was filtered with a 0.22 µm disposable water filter (Biosharp Biotechnology Co., Hefei, China, Cat. No. BS-QT-011) after centrifugation. The column was equilibrated with the mobile phase at a flow rate of 0.8 mL/min for at least 30 min before injection, 30–40 urine samples were tested per day, and the column was rinsed with anhydrous methanol (Analytical grade, Tianjin Komeo Chemical Reagent Co., Tianjin, China) for at least 20 min after testing to ensure good separation performance of the column.

### 2.7. Statistical Analysis

Continuous variables (age, BMI, urinary tAs, urinary iAs, urinary MMA^V^, urinary DMA^V^, iAs%, MMA^V^%, DMA^V^%, PMI, SMI, blood glucose, and serum 25(OH)D) were expressed as mean (±standard deviation) or median (25th percentile-75th percentile, P25-P75). Continuous variables with normal distribution were compared using Student’s *t* test, while continuous variables with non-normal distribution were compared using the Mann–Whitney U test. Categorical variables were compared using the chi-square test. The concentration of different forms of arsenic (As^III^, As^V^, MMA^V^, and DMA^V^) was calculated using a standard curve. iAs and tAs were calculated using the following formula: iAs = As^III^ + As^V^; tAs = iAs + MMA^V^ + DMA^V^. All concentrations of arsenic species in urine were transformed using an extension of the Box–Cox normality transformation for subsequent multiple linear regression analysis. The transformation formula for all concentrations of arsenic species is as follows: y(λ) = (y^λ^ − 1)/λ. The best λ value of all concentrations of arsenic species is as follows: λ (tAs) = 0.298, λ (iAs) = 0.145, λ (MMA^V^) = 0.021, λ (DMA^V^) = 0.330), and the β values in all tables are expressed as Box–Cox transformed β. Multivariable linear regressions were used to evaluate the changes in serum 25(OH)D for each one-unit increment in Box–Cox transformed urinary arsenic species. The multiple linear regression models are as follows:

Model 1: Y = β (tAs or iAs or MMA^V^ or DMA^V^) + ε

Model 2: Y = β (tAs or iAs or MMA^V^ or DMA^V^) + β1 (age) + β2 (gender) + β3 (BMI) + ε

Model 3: Y = β (tAs or iAs or MMA^V^ or DMA^V^) + β1 (age) + β2 (gender) + β3 (BMI) + β4 (occupation) + β5 (education) + β6 (skin hyperkeratosis) + β7 (milk consumption) + β8 (blood glucose) + ε

In the present study, the percentage of people with vitamin D excess is more than 10%, it would be inaccurate to use ORs (odd ratios) to estimate the relative risk. Therefore, we performed multivariate log-binomial regression to assess the relationship between urinary arsenic species and vitamin D status. Vitamin D status was determined using serum 25(OH)D and divided into two categories: normal (30–100 ng/mL) and vitamin D excess (>100 ng/mL) according to the instructions. Six participants with serum 25(OH)D levels below 30 ng/mL were excluded because the sample size was too small for follow-up analysis. The effect estimates were presented as rate ratios (RRs) with their 95% confidence intervals (95% CIs) to estimate the relative risk of vitamin D excess. The multiple linear regression models are as follows:

Model 1: Y = exp (β tAs orβ iAs or β MMA^V^ or β DMA^V^ + ε)

Model 2: Y = exp [β (tAs or iAs or MMA^V^ or DMA^V^) + β1 (gender) + β2 (skin hyperkeratosis) + β3 (milk consumption) + ε]

Stratification of arsenic methylation metabolic capacity by the median of iAs% [(iAs^III^ + iAs^V^)/tAs × 100], MMA^V^% (MMA^V^/tAs × 100), DMA^V^% (DMA^V^/tAs × 100), PMI [MMA^V^ + DMA^V^)/tAs × 100], and SMI [DMA^V^/(MMA^V^ + DMA^V^) × 100]. All statistical analyses were performed in SPSS 26.0 software and a *p*-value < 0.05 (two sides) was considered significant.

## 3. Results

### 3.1. Basic Characteristics of the Study Population

A total of 762 participants were recruited in this study and 86.1% of them were farmers. Of these, 256 participants (33.7%) were male and 506 participants (66.3%) were female. The mean (±SD) age of enrolled participants was 57.92 ± 10.80 years and the mean (±SD) body mass index was 25.77 ± 4.01 kg/m^2^. The median concentrations of urinary tAs, iAs, MMA^V^, and DMA^V^ in urine were 69.81, 3.46, 4.78, and 51.15 μg/L, respectively. Representative chromatograms for arsenic species are shown in Figures S1–S9. The median concentration of blood glucose was 5.70 mmol/L. The mean serum 25(OH)D concentration was 74.03 ± 22.67 ng/mL. These results are presented in Table 1.

### 3.2. Multivariate Linear Regression Analysis between Urinary Arsenic Species and Serum 25(OH)D

Table 2 depicts the association between urinary arsenic species and serum 25(OH)D. For urinary tAs, multivariate linear regression analysis revealed that Box–Cox transformed urinary tAs was positively associated with serum 25(OH)D in the unadjusted model (β = 0.046, 95% CI: 0.020 to 0.071, *p* < 0.01). After we adjusted for age, gender, and BMI in Model 2, we also observed a positive relationship between Box–Cox transformed urinary tAs and serum 25(OH)D (β = 0.044, 95% CI: 0.021 to 0.067, *p* < 0.01). This positive relationship still existed after considering age, gender, BMI, occupation, education, skin hyperkeratosis, milk consumption, and blood glucose in Model 3 (β = 0.044, 95% CI: 0.020 to 0.069, *p* < 0.01).

For urinary iAs, we found a positive association between Box–Cox transformed urinary iAs and serum 25(OH)D in the unadjusted model (β = 0.330, 95% CI: 0.035 to 0.624, *p* < 0.01). However, when we adjusted for other potential confounders, the association between urinary iAs and serum 25(OH)D vanished in Model 2 (β = 0.155, 95% CI: −0.115 to 0.425, *p* = 0.260) and Model 3 (β = 0.100, 95% CI: −0.178 to 0.377, *p* = 0.482).

For urinary MMA^V^, our results showed that Box–Cox transformed urinary MMA^V^ was positively associated with serum 25(OH)D in the unadjusted model (β = 0.447, 95% CI: 0.145 to 0.748, *p* < 0.01). And this positive association still existed after we adjusted for age, gender and BMI (β = 0.276, 95% CI: 0.001 to 0.551, *p* = 0.049). However, when we added potential confounders including occupation, education, skin lesions, and milk consumption, this positive relationship vanished (β = 0.272, 95% CI: −0.013 to 0.556).

For urinary DMA^V^, our multivariate linear regression analysis revealed that Box–Cox transformed urinary DMA^V^ was positively associated with serum 25(OH)D in the unadjusted model (β = 0.057, 95% CI: 0.024 to 0.089, *p* < 0.01). After we adjusted for other potential confounders, this positive association still existed (Model 2: β = 0.060, 95% CI: 0.031 to 0.091, *p* < 0.01; Model 3: β = 0.062, 95% CI: 0.030 to 0.094, *p* < 0.01).

### 3.3. Associations between Urinary Arsenic Species and Vitamin D Status

We excluded six participants with vitamin deficiency [serum 25(OH)D < 30 ng/mL] and included participants with serum 25(OH)D levels of 30–100 ng/mL in the normal group, participants with serum 25(OH)D more than 100 ng/mL were included in the vitamin D excess group. Table S1 shows the basic characteristics of the population between the two groups. No significant difference was found in age, BMI, education, occupation, urinary iAs, urinary MMA^V^ or blood glucose between the two groups (all *p* > 0.05). However, we found that gender, skin hyperkeratosis, and milk consumption varied between the two groups (all *p* < 0.05). We found that the populations in the vitamin D excess group had higher urinary tAs and DMA^V^ levels compared to the normal group (all *p* < 0.05).

Table 3 shows the relationship between urinary arsenic species and vitamin D status. For urinary tAs, our log-binomial regression analysis showed a significant 0.4% increase in the risk of vitamin D excess for each 1 unit increase in Box–Cox transformed urinary tAs in the unadjusted model (RR = 1.004, 95% CI: 1.001 to 1.006, *p* < 0.01). After adjusting for gender, skin health and milk consumption, this positive association remained but was not statistically significant (RR = 1.003, 95% CI: 1.000 to 1.005, *p* = 0.079). For urinary DMA^V^, we found a significant 0.5% increase in the risk of vitamin D excess for each 1-unit increment in Box–Cox transformed urinary DMA^V^ in the unadjusted model (RR = 1.005, 95% CI: 1.001 to 1.008, *p* = 0.011). After adjusting for the above covariates, this positive relationship remained (RR = 1.004, 95% CI: 1.000 to 1.008, *p* = 0.030).

### 3.4. Association between Arsenic Metabolism Efficiency and Serum Vitamin D

We performed linear regression analyses to further identify the effect of arsenic metabolism efficiency on the association between urinary tAs and serum 25(OH)D in different populations (Table 4). Interestingly, no significant correlation was observed between urinary tAs and serum 25(OH)D (β = 0.015, 95% CI: −0.027 to 0.057, *p* = 0.484) in the populations with iAs% below the median. However, in the populations with iAs% above the median, each one-unit increase in the Box–Cox transformed tAs level was associated with increases of 0.064 (95% CI: 0.032 to 0.096, *p* < 0.01) in serum 25(OH)D. We also observed a positive association between urinary tAs and serum 25(OH)D (β = 0.064, 95% CI: 0.032 to 0.096, *p* < 0.01) in the populations with PMI below the median. However, in the populations with PMI above the median, we did not observe a significant correlation between urinary tAs and serum 25(OH)D (β = 0.015, 95% CI: −0.027 to 0.057, *p* = 0.484). These results suggest that the methylation capacity of inorganic arsenic may influence the positive relationship between urinary tAs and serum 25(OH)D levels.

### 3.5. Association between Urinary Arsenic Species and Serum 25 (OH)D in Subgroups Stratified by Skin Hyperkeratosis

The skin is both an organ for vitamin D synthesis and a target organ for inorganic arsenic toxicity. Therefore, stratified analyses were performed to determine the relationship between urinary arsenic species and serum 25(OH)D in populations with normal and arsenic-induced hyperkeratotic skin. Table S2 shows the basic characteristics of the population in two groups. We found that the serum 25(OH)D level was higher in the skin hyperkeratosis group than in the normal group. Table 5 shows the associations between urinary arsenic species and serum 25(OH)D in two groups. In the normal group, positive associations were found between tAs (β = 0.041, 95% CI: 0.013 to 0.069, *p* < 0.01), MMA^V^ (β = 0.425, 95% CI: 0.075 to 0.776, *p* < 0.01) and DMA^V^ (β = 0.054, 95% CI: 0.017 to 0.090, *p* < 0.01) with serum 25(OH)D. However, we only found a positive association between urinary iAs (β = 0.592, 95% CI: 0.041 to 1.143, *p* = 0.035) and serum 25(OH)D in the inorganic arsenic-induced hyperkeratotic skin group.

## 4. Discussion

Vitamin D is a group of biologically active steroid compounds. It occurs naturally as vitamin D2 (ergocalciferol) and vitamin D3 (cholecalciferol) [43,44]. Vitamin D2 is considered a plant form of vitamin D because it is produced by fungi and yeast through exposure to ultraviolet B rays (UVB) [45]. In most vertebrates, 7-dehydrocholesterol is converted into vitamin D3 under UVB rays [46]. When vitamin D2 and vitamin D3 are ingested, both are metabolized in the liver to 25(OH)D. As the main storage form of vitamin D, total 25(OH)D concentrations in serum or plasma reflect skin synthesis, dietary intake and tissue storage of vitamin D. Therefore, 25(OH)D is often considered as a reliable biomarker of nutritional status of the endocrine vitamin D system [47]. Experts believe that the lower limit of adequate 25(OH)D levels should be 30 ng/mL [9]. Excess vitamin D was defined when serum 25(OH)D levels exceeded about 100 ng/mL [8]. When serum 25(OH)D levels are higher than 150 ng/mL, symptoms related to vitamin D poisoning will occur, such as confusion, apathy, repeated vomiting, abdominal pain, polyuria, polydipsia, and dehydration [11].

There are few studies on the relationship between urinary arsenic and serum vitamin D. A prospective cohort study focusing on the association between maternal metal (loid) exposure during pregnancy with newborns’ vitamin D status in China found a positive correlation between urinary tAs and serum 25(OH)D [27]. In Torreón, a city in Mexico, a case–control study focusing on adolescents found no association between urinary tAs and serum 25(OH)D, but urinary tAs was positively correlated with 1,25(OH)_2_D, the active form of vitamin D [26]. In the present study, we analyzed the association of urinary tAs with serum 25(OH)D. Although arsenobtaine, a nontoxic arsenic, can be detected in the urine of people consuming seafood, arsenobetaine is not metabolized to inorganic arsenic in the bloodstream to a significant degree [48]. Therefore, we excluded participants with recent seafood consumption and used iAs, MMA^V^, and DMA^V^ as the primary basis for calculating tAs. We observed a positive correlation between urinary tAs and serum 25(OH)D in all linear regression models, and in the log-binomial regression analysis, urinary tAs was positively associated with the risk of vitamin D excess. In another in vitro study, As^III^ inactivated the mitochondrial enzyme 24-hydroxylase to increase the activity of paricalcitol, an analogue of 1,25(OH)_2_D, in acute myeloid leukemia cells [25]. Therefore, exposure to inorganic arsenic may affect the process of vitamin D metabolism, but the exact mechanism still needs to be investigated. In addition, we found that there were positive associations between urinary DMA^V^ and serum 25(OH)D in all linear regression models and that DMA^V^ was positively associated with the risk of vitamin D excess in the log-binomial regression analysis. Our results suggest that the efficiency of inorganic arsenic metabolism may influence serum 25(OH)D levels.

Once inorganic arsenic is ingested, it will be metabolized in the liver. As^III^ accepts a methyl group from S-adenosylmethionine and is metabolized to MMA^V^ and DMA^V^ through a series of oxidation and methylation processes [24,49]. These arsenic species (iAs, MMA^V^, and DMA^V^) are excreted in urine and can be measured and expressed as a percentage of total urinary arsenic (iAs %, MMA^V^ %, and DMA^V^ %) [50]. In general, a low iAs% indicates increased methylation capacity, while a high iAs % indicates decreased methylation capacity. It is believed that increased methylation capacity may reduce susceptibility to inorganic arsenic-related toxicity [50,51,52]. The results in the present study showed a significant positive correlation between urinary tAs and serum 25(OH)D in the populations with iAs % above the median and PMI below the median. However, no significant correlation was found between urinary tAs and serum 25(OH)D in the populations with iAs % below the median and PMI above the median. Therefore, our study established a positive association between urinary tAs and serum 25(OH)D in the incompletely methylated population and this association might be modified by the efficiency of inorganic arsenic metabolism. However, further work is needed to expand the sample size for relevant studies.

The maintenance of skin structure depends on the balance between differentiation and proliferation of keratinocytes. Chronic exposure to inorganic arsenic dramatically disrupts this balance and ultimately leads to hyperkeratosis of the skin [41,53,54]. The epidermis is also the main source of vitamin D for the body, as the keratinocytes in the epidermis are capable of producing 25(OH)D3 [38,55,56]. The results of our stratified analyses showed a positive association between urinary iAs and serum 25(OH)D in the arsenic-induced hyperkeratotic skin group. Future work is needed to explore the mechanisms.

A limitation of our study is its cross-sectional nature, which restricts inferences about causality, and further longitudinal studies are needed. Second, we did not collect information on time spent outdoors because solar radiation is a confounding factor, but our study population was homogeneous, 86.1% of them were farmers and their lifestyles were similar. Third, the present study examined the concentrations of iAs, MMA^V^, and DMA^V^ in urine samples, but the presence of MMA^III^ and DMA^III^ in human urine samples cannot be excluded. A previous study explored the retention behavior of As^III^, As^V^, MMA^III^, DMA^III^, MMA^V^, and DMA^V^ on PRP-X100 anion-exchange columns over the pH range of 5–9. The results indicated that urinary DMA^III^ is likely to be strongly retained on this analytical system and at the chosen mobile phase pH of this study, while MMA^III^ may have co-eluted with MMA^V^ [57]. This is a flaw in our study (sometimes the broad MMA^V^ peaks that were observed in the urine analysis seen in Figures S1–S9 support a likely co-elution of MMA^III^ and MMA^V^). Based on the higher toxicity of MMA^III^ and DMA^III^ than inorganic arsenic, we recommend evaluating the association of MMA^III^ and DMA^III^ with serum vitamin D in future studies. Finally, further in vitro experiments are recommended to evaluate whether inorganic arsenic leads to increased vitamin D levels by promoting the hyperproliferation of keratinocytes.

## 5. Conclusions

In this study, we found that urinary DMA^V^ was positively associated with serum 25(OH)D. This suggests that excessive inorganic arsenic exposure may affect vitamin D metabolism with the exact mechanism still needing to be elucidated. We recommend appropriate vitamin D supplementation to people living in areas with water-borne arsenicosis to avoid vitamin D poisoning.

## Figures and Tables

**Table 1:** Basic characteristics of study subjects.

Characteristics	Value
Age, years, mean ± SD	57.92 ± 10.80
BMI, kg/m^2^, mean ± SD	25.77 ± 4.01
Gender, n (%)	
Male	256 (33.7)
Female	506 (66.3)
Skin hyperkeratosis	
No	495 (64.9)
Yes	267 (35.1)
Occupation, n (%)	
Farmer	657 (86.1)
Others	105 (13.9)
Education, n (%)	
Primary and below	269 (35.4)
Junior high school	409 (53.6)
Senior high school and above	84 (11)
Milk consumption, n (%)	
≤1/week	471 (61.8)
>1/week	291 (38.2)
Urinary tAs, μg/L, median (P25–P75)	69.81 (27.77–137.51)
Urinary iAs, μg/L, median (P25–P75)	3.46 (0.83–14.67)
Urinary MMA^V^, μg/L, median (P25–P75)	4.78 (0.32–16.14)
Urinary DMA^V^, μg/L, median (P25–P75)	51.15 (19.00–102.93)
Urinary iAs%, median (P25–P75)	9.94 (2.39–18.58)
Urinary MMA^V^%, median (P25–P75)	9.41 (1.60–16.88)
Urinary DMA^V^%, median (P25–P75)	78.48 (66.53–90.18)
PMI, median (P25–P75)	89.81 (81.26–97.51)
SMI, median (P25–P75)	89.15 (80.02–97.93)
Blood glucose, mmol/L, median (P25–P75)	5.70 (5.10–6.70)
25(OH)D, ng/mL, mean ± SD	74.03 ± 22.67

**Table 2:** Association between urinary arsenic species and serum 25(OH)D.

Exposure	Box–Cox Transformed β (95% CI)	*p*-Value
tAs		
Model 1	0.046 (0.020, 0.071)	<0.01
Model 2	0.044 (0.021, 0.067)	<0.01
Model 3	0.044 (0.020, 0.069)	<0.01
iAs		
Model 1	0.330 (0.035, 0.624)	0.028
Model 2	0.155 (−0.115, 0.425)	0.260
Model 3	0.100 (−0.178, 0.377)	0.482
MMA^V^		
Model 1	0.447 (0.145, 0.748)	<0.01
Model 2	0.276 (0.001, 0.551)	0.049
Model 3	0.272 (−0.013, 0.556)	0.061
DMA^V^		
Model 1	0.057 (0.024, 0.089)	<0.01
Model 2	0.060 (0.031, 0.091)	<0.01
Model 3	0.062 (0.030, 0.094)	<0.01

Model 1: non-adjusted. Model 2: adjusted for age, gender, and BMI. Model 3: adjusted for age, gender, BMI, occupation, education, skin hyperkeratosis, milk consumption and blood glucose.

**Table 3:** Association between urinary arsenic species and vitamin D status.

Exposure	Box–Cox Transformed β	Box–Cox Transformed RR (95% CI)	*p*-Value
tAs			
Model 1	0.004	1.004 (1.001, 1.006)	0.016
Model 2	0.003	1.003 (1.000, 1.005)	0.079
iAs			
Model 1	0.020	1.020 (0.987, 1.054)	0.240
Model 2	0.002	1.002 (0.974, 1.302)	0.869
MMA^V^			
Model 1	0.022	1.022 (0.988, 1.057)	0.200
Model 2	0.004	1.004 (0.973, 1.037)	0.784
DMA^V^			
Model 1	0.005	1.005 (1.001, 1.008)	0.011
Model 2	0.004	1.004 (1.000, 1.008)	0.030

Model 1: non-adjusted. Model 2: adjusted for gender, skin hyperkeratosis and milk consumption.

**Table 4:** Multivariable regression analyses of the associations between urinary tAs and serum 25(OH)D stratified for arsenic metabolism efficiency.

	Box–Cox Transformed β (95% CI) ^b^	*p*-Value ^b^
iAs% ^a^		
<10.19	0.015 (−0.027, 0.057)	0.484
≥10.19	0.064 (0.032, 0.096)	<0.01
MMA^V^% ^a^		
<9.54	0.053 (0.012, 0.094)	0.036
≥9.54	0.038 (0.006, 0.070)	0.044
DMA^V^% ^a^		
<78.15	0.046 (0.013, 0.078)	<0.01
≥78.15	0.047 (0.006, 0.087)	0.027
PMI		
<89.81	0.064 (0.032, 0.096)	<0.01
≥89.81	0.015 (−0.027, 0.057)	0.484
SMI		
<89.15	0.041 (0.009, 0.073)	0.013
≥89.15	0.050 (0.009, 0.091)	0.017

^a^ Stratified for median of each metabolite. ^b^ The multiple regression value without adjusting factors.

**Table 5:** Association between urinary arsenic species and serum 25(OH)D by skin hyperkeratosis stratified.

Box–Cox Transformed β (95% CI) ^a^				
Exposure	Normal	*p*-Value	Skin Hyperkeratosis	*p*-Value
tAs	0.041 (0.013, 0.069)	<0.01	0.046 (−0.007, 0.099)	0.090
iAs	0.169 (−0.173, 0.511)	0.332	0.592 (0.041, 1.143)	0.035
MMA^V^	0.425 (0.075, 0.776)	<0.01	0.430 (−0.139, 0.999)	0.138
DMA^V^	0.054 (0.017, 0.090)	<0.01	0.052 (−0.015, 0.119)	0.129

^a^ The multiple regression value without adjusting factors.

## Data Availability

Data are contained within the article.

## Supplementary Materials

The following supporting information can be downloaded at: https://www.mdpi.com/article/10.3390/toxics12010083/s1, Figures S1–S9: Representative HPLC-AFS chromatograms. Table S1: Population’s characteristics description of the normal group and vitamin D excess group; Table S2: Population’s characteristics description of the normal group and skin hyperkeratosis group.
