# Supplementary material for: Positive Association of Urinary Dimethylarsinic Acid (DMAV) with Serum 25(OH)D in Adults Living in an Area of Water-Borne Arsenicosis in Shanxi, China
Source: Toxics. 2024 Jan 18;12(1):83. doi: 10.3390/toxics12010083 (PMC10820359; doi:10.3390/toxics12010083)
Supplement: Supplementary file 1 [file toxics-12-00083-s001.zip › Supplemental materials.pdf]

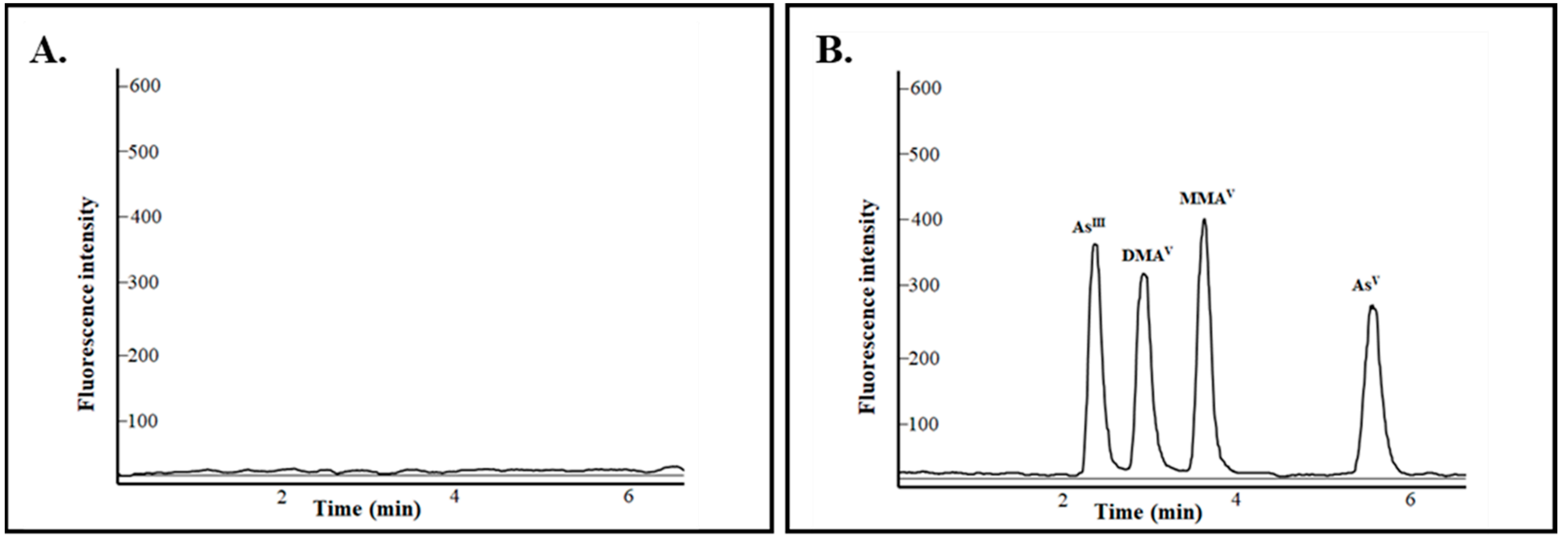

Figure S1. Representative HPLC-AFS chromatograms. (A) Representative HPLC-AFS chromatogram of blank. (B) Representative HPLC-AFS chromatogram of the standards, with concentrations recorded as As at 10 ng/mL (As^{III}, DMA^{V}, MMA^{V}, and As^{V} concentrations are all 10 ng/mL).

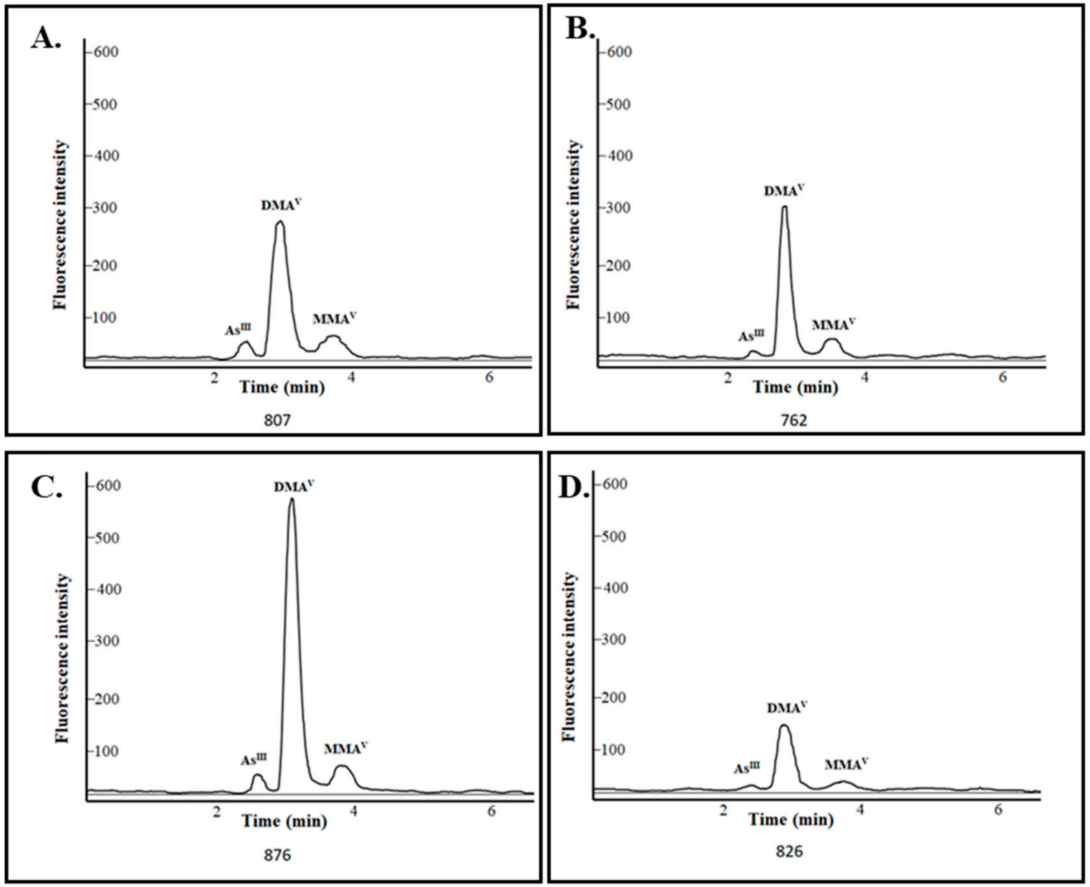

Figure S2. Representative HPLC-AFS chromatograms. (A) Representative HPLC-AFS chromatogram of urine sample #807. (B) Representative HPLC-AFS chromatogram of urine sample #762. (C) Representative HPLC-AFS chromatogram of urine sample #876. (D) Representative HPLC-AFS chromatogram of urine sample #826.

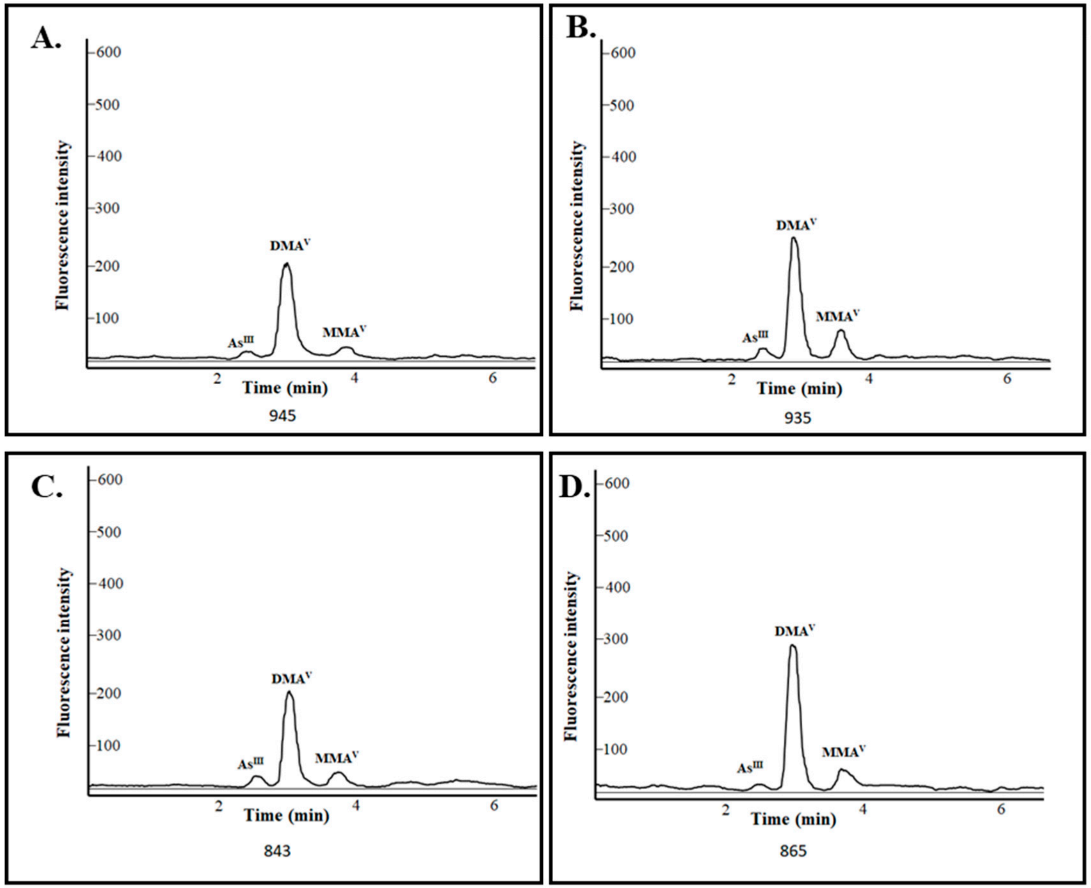

Figure S3. Representative HPLC-AFS chromatograms. (A) Representative HPLC-AFS chromatogram of urine sample #945. (B) Representative HPLC-AFS chromatogram of urine sample #935. (C) Representative HPLC-AFS chromatogram of urine sample #843. (D) Representative HPLC-AFS chromatogram of urine sample #865.

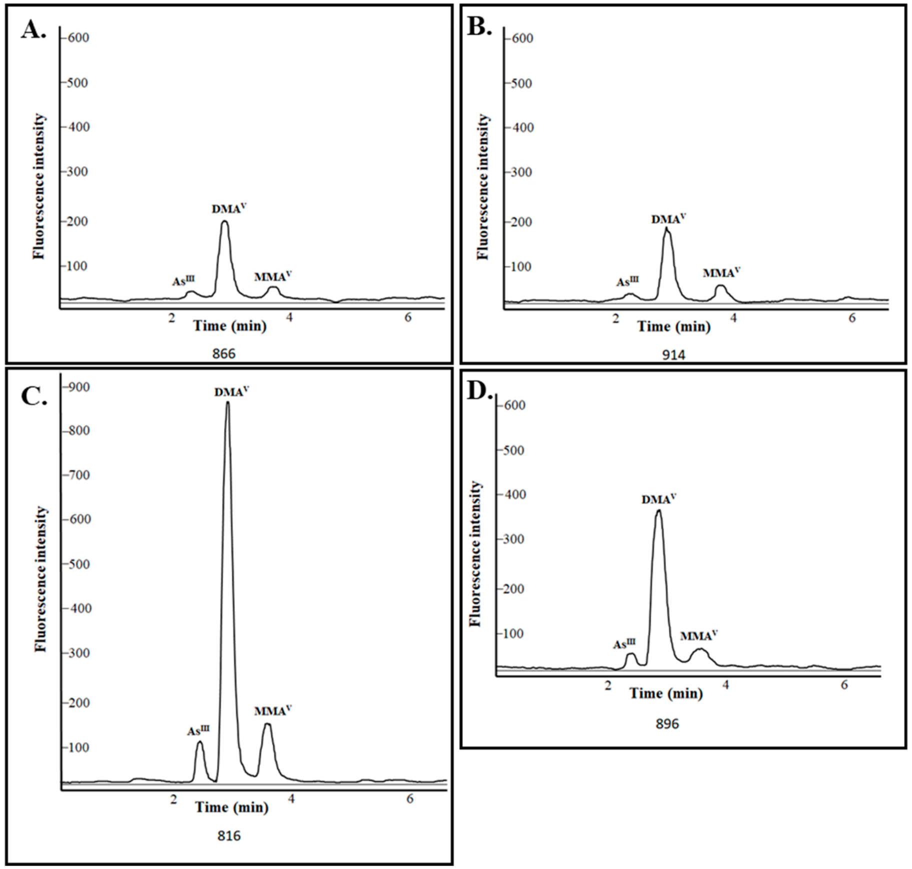

Figure S4. Representative HPLC-AFS chromatograms. (A) Representative HPLC-AFS chromatogram of urine sample #866. (B) Representative HPLC-AFS chromatogram of urine sample #914. (C) Representative HPLC-AFS chromatogram of urine sample #816. (D) Representative HPLC-AFS chromatogram of urine sample #896.

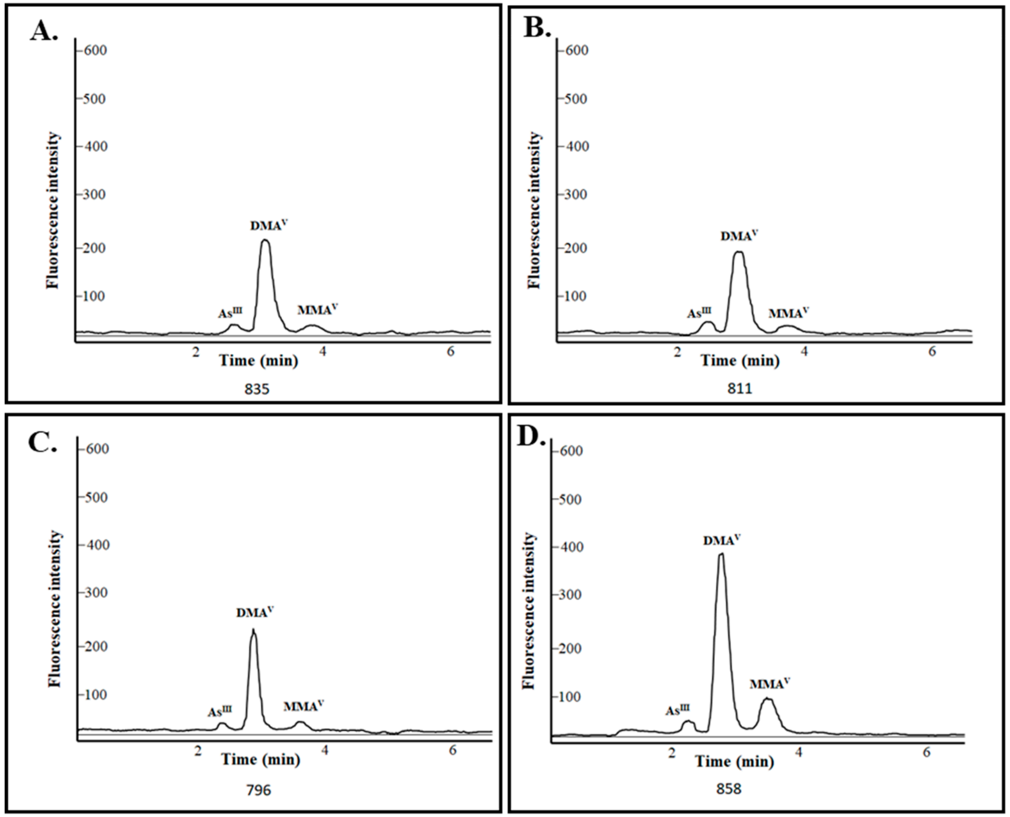

Figure S5. Representative HPLC-AFS chromatograms. (A) Representative HPLC-AFS chromatogram of urine sample #835. (B) Representative HPLC-AFS chromatogram of urine sample #811. (C) Representative HPLC-AFS chromatogram of urine sample #796. (D) Representative HPLC-AFS chromatogram of urine sample #858.

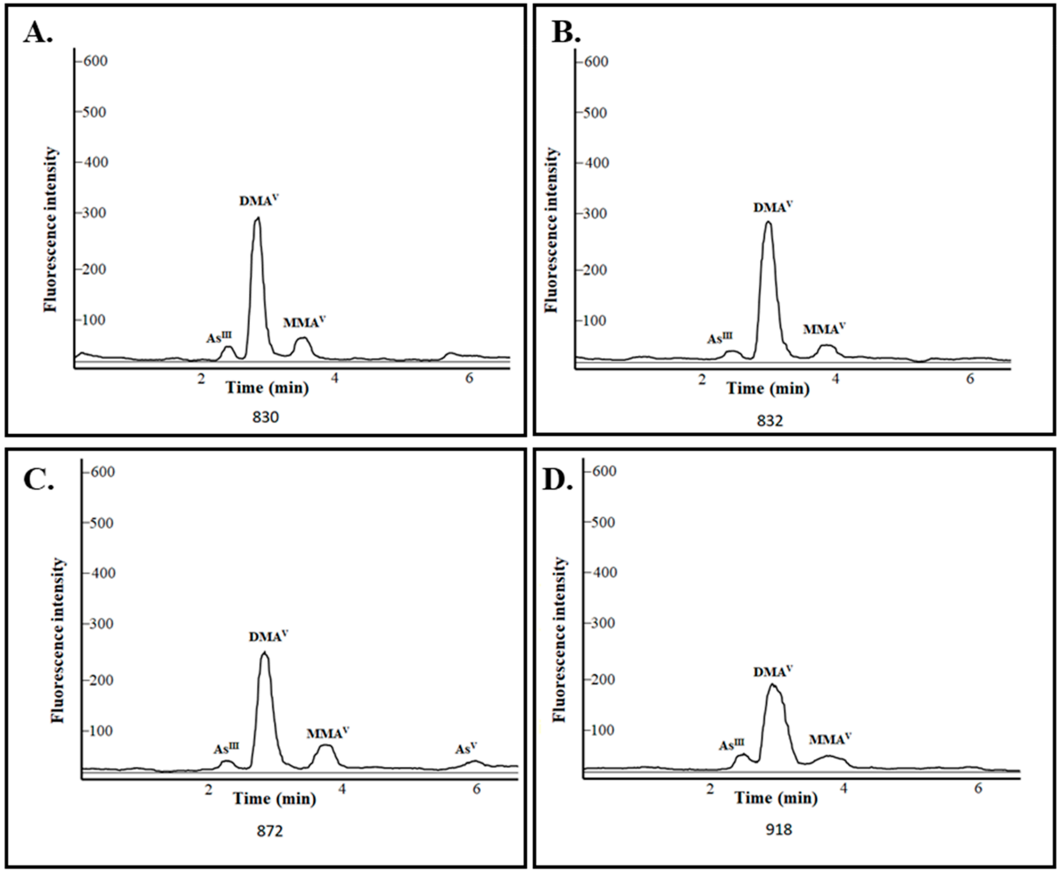

Figure S6. Representative HPLC-AFS chromatograms. (A) Representative HPLC-AFS chromatogram of urine sample #830. (B) Representative HPLC-AFS chromatogram of urine sample #832. (C) Representative HPLC-AFS chromatogram of urine sample #872. (D) Representative HPLC-AFS chromatogram of urine sample #918.

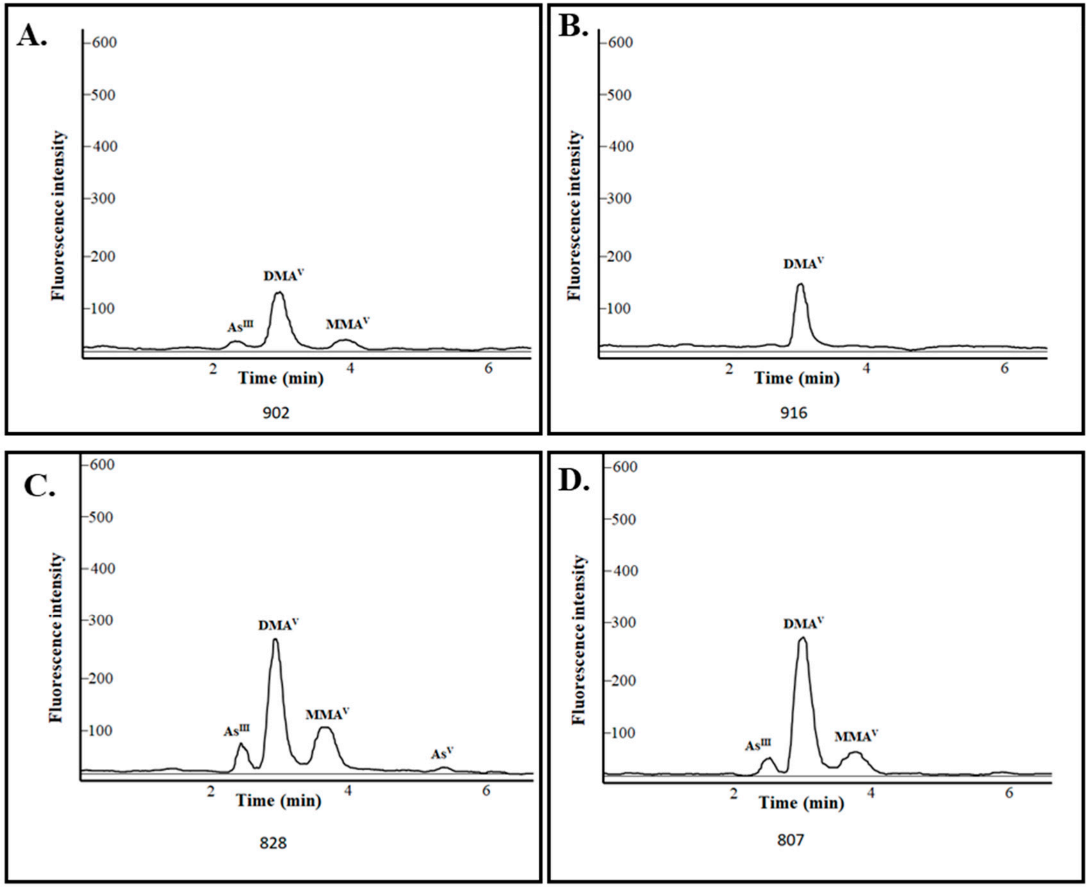

Figure S7. Representative HPLC-AFS chromatograms. (A) Representative HPLC-AFS chromatogram of urine sample #902. (B) Representative HPLC-AFS chromatogram of urine sample #916. (C) Representative HPLC-AFS chromatogram of urine sample #828. (D) Representative HPLC-AFS chromatogram of urine sample #807.

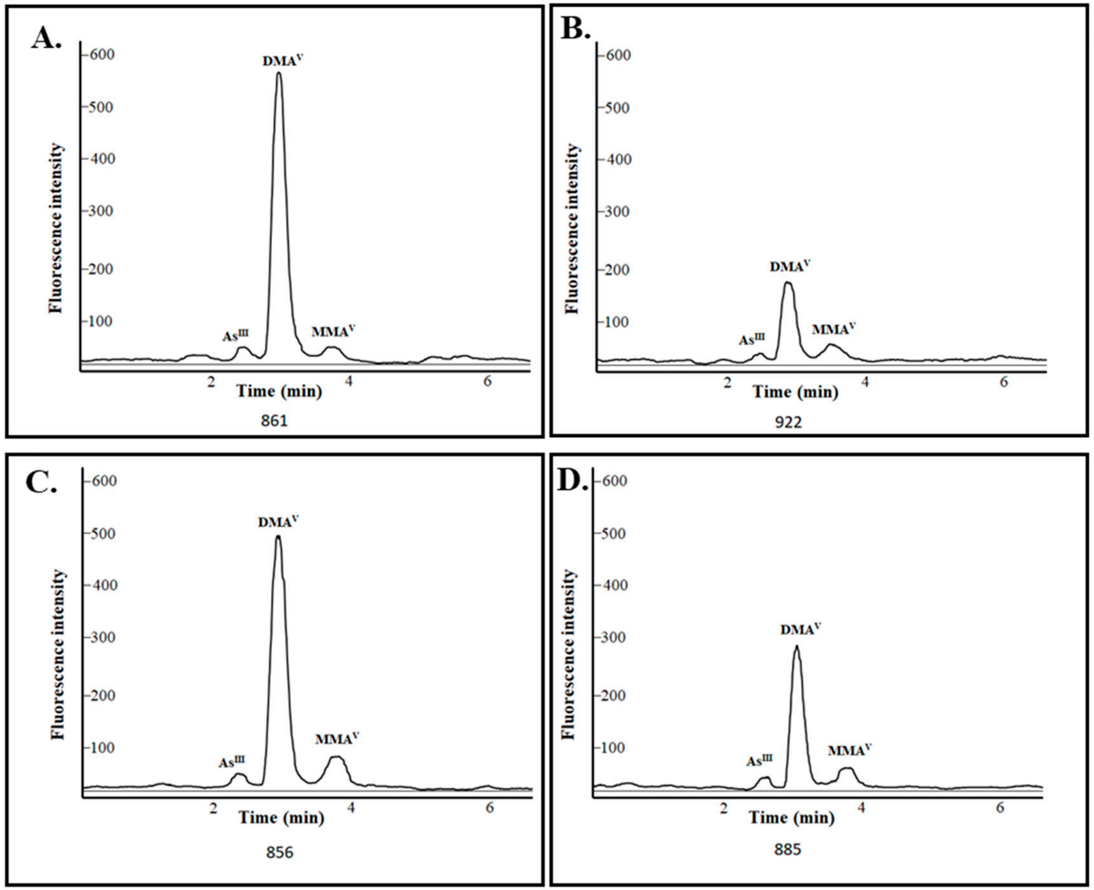

Figure S8. Representative HPLC-AFS chromatograms. (A) Representative HPLC-AFS chromatogram of urine sample #861. (B) Representative HPLC-AFS chromatogram of urine sample #922. (C) Representative HPLC-AFS chromatogram of urine sample #856. (D) Representative HPLC-AFS chromatogram of urine sample #885.

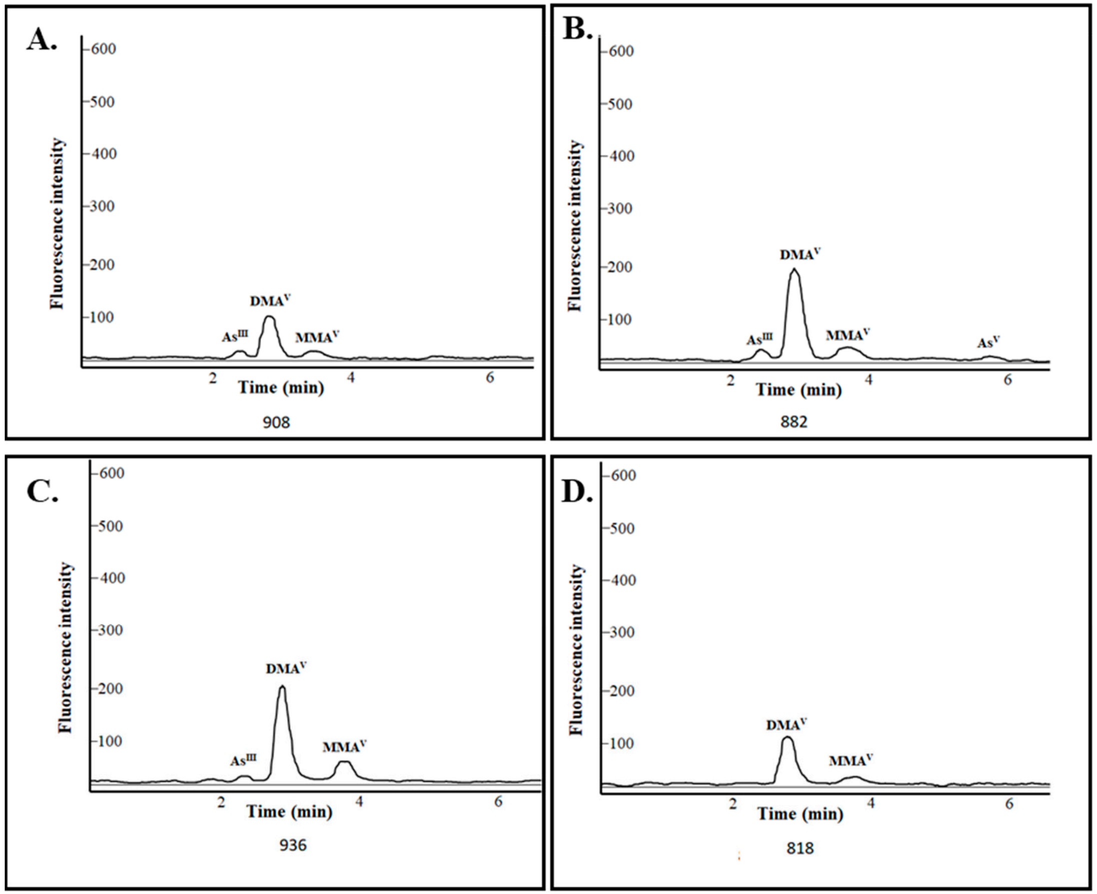

Figure S9. Representative HPLC-AFS chromatograms. (A) Representative HPLC-AFS chromatogram of urine sample #908. (B) Representative HPLC-AFS chromatogram of urine sample #882. (C) Representative HPLC-AFS chromatogram of urine sample #936. (D) Representative HPLC-AFS chromatogram of urine sample #818.

Table S1 Population's characteristics description of the normal group and vitamin D excess group

Variables	Normal group	Vitamin D excess group	t/χ2/z	*P-Value*
	Mean ± SD/median (P25-P75)/n (%)			
Age, years, mean ± SD	57.58 ± 10.80	59.47 ± 10.79	-1.709	0.088
BMI, kg/m2, mean ± SD	25.88 ± 3.70	25.16 ± 5.48	1.763	0.078
Gender, n (%)				
Male	175 (23.1)	81 (10.7)	86.83	**< 0.01**
Female	469 (62.0)	31 (4.2)		
Skin hyperkeratosis				
No	426 (56.3)	63 (8.3)	4.093	**0.043**
Yes	218 (28.8)	49 (6.6)		
Education, n (%)				
Primary and below	221 (29.2)	44 (5.8)	1.083	0.582
Junior high school	353 (46.7)	56 (7.4)		
Senior high and above	70 (9.3)	12 (1.6)		
Occupation, n (%)				
Farmer	551 (72.8)	101 (13.4)	1.716	0.190
Others	93 (12.3)	11 (1.5)		
Milk consumption, n (%)				
>1/week	256 (33.9)	32 (4.2)	5.057	**0.025**
≤1/week	388 (51.3)	80 (10.6)		
Urinary tAs, μg/L, median (P25-P75)	66.94 (24.98-132.63)	84.38 (37.59-152.59)	-2.430	**0.015**
Urinary iAs, μg/L, median (P25-P75)	3.19 (0.83-14.35)	6.57 (0.83-17.47)	-1.793	0.073
Urinary MMA^V, μg/L, median (P25-P75)	4.33 (0.32-15.07)	6.84 (0.32-19.11)	-1.490	0.136
Urinary DMA^V, μg/L, median (P25-P75)	48.59 (17.96-100.88)	70.85 (32.13-122.89)	-2.555	**0.011**

Blood glucose, mmol/L, median (P25-P75)	5.80 (5.10, 7.35)	5.70 (5.10, 6.70)	-1.088	0.276

Table S2 Population's characteristics description of the normal group and skin hyperkeratosis group

Variables	Normal group	Skin hyperkeratosis group	t/χ2/z	*P-Value*
	Mean ± SD/median (P25-P75)/n (%)			
Age, years, mean ± SD	58.25 ± 11.08	57.30 ± 10.26	1.150	0.250
BMI, kg/m2, mean ± SD	25.59 ± 4.19	26.09 ± 3.63	-1.620	0.106
Gender, n (%)				
Male	166 (21.8)	90 (11.8)	0.002	0.962
Female	329 (43.2)	177 (23.2)		
Education, n (%)				
Primary and below	185 (24.3)	84 (11.0)	4.347	0.114
Junior high school	252 (33.1)	157 (20.6)		
Senior high and above	58 (7.6)	26 (3.4)		
Occupation, n (%)				
Farmer	423 (55.5)	234 (30.7)	0.698	0.404
Others	72 (9.5)	33 (4.3)		
Milk consumption, n (%)				
>1/week	185 (24.3)	106 (13.9)	0.398	0.528
≤1/week	310 (40.7)	161 (21.1)		
Urinary tAs, μg/L, median (P25-P75)	57.49 (19.92-122.79)	80.04 (41.42-149.22)	-3.917	**< 0.01**
Urinary iAs, μg/L, median (P25-P75)	3.19 (0.83-14.47)	5.28 (0.83-15.10)	-0.836	0.403
Urinary MMA^V, μg/L, median (P25-P75)	3.58 (0.32-15.34)	6.85 (0.32-17.30)	-1.700	0.089
Urinary DMA^V, μg/L, median (P25-P75)	45.32 (14.08-95.58)	66.70 (32.70-119.50)	-4.044	**< 0.01**
Blood glucose, mmol/L, median (P25-P75)	5.80 (5.10, 6.70)	5.60 (5.10, 6.60)	-1.687	0.092
25(OH)D, ng/mL, mean ± SD	72.79 ± 21.14	76.34 ± 25.15	-2.064	**0.039**
